# Urgent call for compulsory premarital screening: a crucial step towards thalassemia prevention in Bangladesh

**DOI:** 10.1186/s13023-024-03344-1

**Published:** 2024-09-06

**Authors:** Md. Jubayer Hossain, Manisha Das, Ummi Rukaiya Munni

**Affiliations:** 1 Population Health Studies Division, Center for Health Innovation, Research, Action, and Learning – Bangladesh (CHIRAL Bangladesh), 100, Shukrabad, Mirpur Road, Dhanmondi, 1207 Dhaka, Bangladesh; 2 Dhaka Medical College and Hospital, Secretariat Rd, Dhaka, 1000 Bangladesh; 3 International Centre for Diarrhoeal Disease Research, Bangladesh, 68, Shaheed Tajuddin Ahmed Sarani Mohakhali, 1212 Dhaka, Bangladesh

**Keywords:** Premarital screening, Thalassemia prevention, Compulsory screening, Urgent call, Bangladesh

## Abstract

Thalassemia poses a major public health concern in Bangladesh with a high prevalence of carriers. However, there is a substantial knowledge gap regarding its epidemiology, clinical aspects, and treatment outcomes. Despite its high prevalence, there is a notable lack of awareness regarding thalassemia in the Bangladeshi population. The absence of precisely validated data impedes a comprehensive understanding of this disease.

Premarital thalassemia screening is reportedly a successful strategy for countries such as Saudi Arabia and Iran and has also been proposed for Bangladesh. Mandatory screening coupled with genetic counseling is promising for reducing the prevalence of thalassemia by identifying carriers and providing relevant health education. However, sociocultural barriers, challenges, financial constraints, and health risks associated with prenatal diagnosis and abortion could hinder the success of such programs.

Positive outcomes from other countries underscore the effectiveness of such programs in reducing thalassemia incidence. The early identification of carriers and genetic counseling can significantly reduce the burden of thalassemia. Additionally, the strain on the healthcare system would be eased, and the quality of life of thalassemia patients would be improved.

In conclusion, based on evidence mandatory premarital screening with genetic counseling could be an effective measure to reduce the prevalence of thalassemia in Bangladesh. Leveraging positive attitudes, adopting successful international models, and addressing existing challenges are crucial for the successful implementation of programs that contribute to the overall health and well-being of the country’s population.

## Letter to the Editor

Thalassemia is a major public health concern in Bangladesh. Bangladesh is located in the presumed thalassemia belt, with a large number of carriers of thalassemia and hereditary hemoglobinopathies [1, 2]. An estimation using limited prevalence data showed that approximately 6–12% of the population are carriers [3]. Furthermore, a study in Sylhet, Bangladesh, found that 11% of women and 10% of children had an inherited blood disorder, including alpha or beta thalassemia, hemoglobin E, hemoglobin S, hemoglobin D, and other abnormalities [3, 4].

Despite the high prevalence of thalassemia, there is a lack of definitive national data on various aspects, including epidemiology, clinical course, mortality, complications, and treatment outcomes, which has hindered a comprehensive understanding of the disease burden in Bangladesh [1]. Moreover, studies have revealed that despite the higher prevalence of thalassemia carriers, there is a lack of awareness and knowledge about the disease among the population, including university students and indigenous communities [5, 6]. Nonetheless, based on the available data, thalassemia is a potentially rising health concern for Bangladeshi people. This highlights the need for further research and public health interventions to evaluate the current situation and scenario and address the burden of thalassemia.

Premarital screening for thalassemia has been implemented in several countries, including the Middle East (General), Saudi Arabia, Iran, Pakistan, China, Turkey, and Jordan, to reduce the prevalence of thalassemia and other inherited hematological disorders [7–14]. The success of such programs has been attributed to mandatory screening, resulting in a significant reduction in its prevalence [7, 15]. Many countries have reported high levels of community awareness and participation in premarital screening programs. For instance, in Saudi Arabia, a significant portion of the community supported and agreed to undergo elective premarital screening for thalassemia [16]. Premarital screening programs in Saudi Arabia have shown varying success rates in identifying high-risk couples and reducing the incidence of serious genetic illnesses, such as thalassemia. While some studies indicate a significant reduction in the detection of at-risk marriages by approximately 60% [15], others suggest that a large proportion of high-risk couples continue to marry despite receiving genetic counseling [7, 17]. However, over time, regions such as the eastern part of Saudi Arabia have demonstrated a decline in at-risk marriages, indicating the positive impact of premarital screening initiatives [18]. In countries such as Iran, Turkey, and Iraq, the implementation of premarital screening programs has been associated with substantial reductions in the number of births of thalassemia major babies, indicating the effectiveness of these programs in disease prevalence control [9]. Screening programs have been effective in identifying carriers of thalassemia and at-risk couples. For example, in Denizli, Turkey, carrier couples were identified and provided with options for prenatal diagnosis and decision making [11]. In Turkey, a premarital screening program in Denizli found that 2.6% of screened individuals had the β-thalassemia trait, and in 15 out of 9,902 couples, both partners were carriers. After counseling, 2 of the 15 planned carrier marriages were canceled [11]. In Iran, Turkey, and Iraq, reductions of at least 65% in thalassemia affected births have been reported after the implementation of premarital screening programs [9]. Southern Turkey’s premarital hemoglobinopathy screening program resulted in a 90% reduction in affected births over 10 years [19]. In Saudi Arabia, among the high-risk couples identified through premarital screening, 89.6% chose to marry each other, despite their known risk status [20]. In Denizli, Turkey, following genetic counseling, only 2 out of 15 high-risk couples canceled their marriage plans [11]. Moreover, by enforcing a pre-marital screening law in Cyprus, the incidence decreased to 90% [21].

The success of premarital screening programs is also influenced by factors such as public awareness, knowledge, and attitudes towards genetic disorders. Studies have shown varying levels of awareness among individuals, with some indicating a lack of knowledge about thalassemia among college students in Saudi Arabia [22] and premarital couples in Indonesia, while others highlight a higher awareness level among parents of children with thalassemia in Pakistan. Some regions, such as Punjab, Pakistan, face challenges in implementation, including a lack of infrastructure, cultural beliefs, and limited public awareness, which suggests the need for comprehensive strategies beyond screening [23]. Additionally, studies have reported that health education and counseling play crucial roles in improving knowledge and attitudes towards premarital screening programs [24–26]. In Bangladesh, compulsory premarital screening, coupled with genetic counseling, has been proposed as an effective strategy to prevent thalassemia [3, 15, 16]. However, socioeconomic challenges, religious beliefs, and health risks associated with prenatal diagnosis have been highlighted as potential barriers to the success of such programs [5, 27–30].

In addition to mandatory premarital screening programs, studies have shown that voluntary screening programs are effective in increasing awareness, early detection, and prevention of genetic diseases such as thalassemia [9, 31]. These initiatives allow individuals to proactively assess their genetic risks and make informed decisions regarding marriage and family planning. The success of such voluntary screening programs has been observed in countries such as Iran, Pakistan, Italy and Greece [31–33]. By adopting a life cycle approach to thalassemia prevention and intervention at critical periods during the youth, premarital, prenatal, and neonatal stages, voluntary screening has been shown to complement premarital screening efforts and enhance the overall effectiveness of thalassemia prevention strategies [9]. A long-term voluntary screening program is highly effective in Sardinia. It achieved a reduction in the birth rate of thalassemia major from 1:250 live births to 1:4000. This success was attributed to the voluntary screening offered to prospective parents and education of the population, along with the training of health personnel [32]. The National Thalassemia Prevention Programmer in Northern Greece, over a 15-year period (2001–2015), demonstrated a significant decrease in the incidence of affected newborns, achieving a 90% reduction in new affected births. This program effectively incorporates carrier screening and genetic counseling [33]. Voluntary screening programs, when combined with prenatal diagnosis, have shown success in different cultural and social contexts. In both Italy and Greece, these programs have led to significant reductions in thalassemia major births, demonstrating their potential effectiveness in various settings [32]. Evidence from Italy and Greece supports the effectiveness of voluntary thalassemia prevention programs, which include components of public education, carrier screening, genetic counseling, and prenatal diagnosis. These findings suggest that a similar voluntary approach, appropriately adapted to the cultural and social context of Bangladesh, could be a viable alternative to mandatory premarital screening.

Since most people in Bangladesh have good attitudes regarding premarital screening, there could be a significant impact on lowering the prevalence of thalassemia if this practice is made mandatory [3, 5, 28, 29]. By identifying carrier couples and providing them with genetic counseling, the incidence of thalassemia major can be significantly reduced, thereby alleviating the burden on the healthcare system and improving the quality of life of affected individuals [15, 34, 35].

In conclusion, compulsory premarital screening coupled with genetic counseling has the potential to significantly reduce the prevalence of thalassemia in Bangladesh. By leveraging the positive attitudes of the population towards premarital screening and drawing from the success of similar programs in other countries, Bangladesh can make substantial strides in preventing thalassemia and improving public health. However, it is essential to address existing challenges and barriers to ensure the effectiveness of such preventive measures.

## Data Availability

This study is a synthesis of existing research and does not include any new or third-party data. All information was derived from previously published sources, which are cited in the references section.
